# GETTING TO BOLD USING SYSTEMIC EDUCATION, EVIDENCE, AND NETWORKS: HELPING GEORGIANS B-SEEN

**DOI:** 10.1093/geroni/igac059.513

**Published:** 2022-12-20

**Authors:** Elizabeth Head

**Affiliations:** Georgia Department of Public Health, Atlanta, Georgia, United States

## Abstract

The complex nature of Alzheimer’s disease and related dementias (ADRD) demands a comprehensive public health approach. Georgia is Building Our Largest Dementia infrastructure using Systemic Education, Evidence, and Networks (B-SEEN), with a vision for every Georgian – professional, patient, and care partner – to B-SEEN. The strength of Georgia’s B-SEEN project is the existing infrastructure. Leveraging this established network, Georgia has engaged in population-based efforts to increase impact in the dementia risk reduction, early diagnosis of ADRD, prevention and management of comorbidities and avoidable hospitalizations, and caregiving. These outcomes are being achieved by stakeholders disseminating evidence-based programs personalized to their community needs and the B-SEEN team leading coordinated activities that address dementia and support the promotion of brain health. This presentation will describe how ADRD risk reduction is integrated into a 159 county, de-centralized state and provides examples of several activities being implemented via Georgia extension, faith-based organizations, and dedicated partners.

